# A novel thiol-saccharide mucolytic for the treatment of muco-obstructive lung diseases

**DOI:** 10.1183/13993003.02022-2022

**Published:** 2023-05-25

**Authors:** Annalisa Addante, Wilfred Raymond, Irina Gitlin, Annabelle Charbit, Xavier Orain, Aaron Wolfe Scheffler, Aditi Kuppe, Julia Duerr, Maria Daniltchenko, Marika Drescher, Simon Y. Graeber, Anne-Marie Healy, Stefan Oscarson, John V. Fahy, Marcus A. Mall

**Affiliations:** 1 Department of Pediatric Respiratory Medicine, Immunology and Critical Care Medicine, Charité – Universitätsmedizin Berlin, corporate member of Freie Universität Berlin and Humboldt-Universität zu Berlin, Berlin, Germany; 2 German Centre for Lung Research (DZL), associated partner, Berlin, Germany; 3 Cardiovascular Research Institute, University of California San Francisco, San Francisco, CA, USA; 4 Department of Epidemiology and Biostatistics, University of California San Francisco, San Francisco, CA, USA; 5 Berlin Institute of Health (BIH) at Charité – Universitätsmedizin Berlin, Berlin, Germany; 6 School of Pharmacy and Pharmaceutical Sciences, Trinity College Dublin, Dublin, Ireland; 7 Centre for Synthesis and Chemical Biology, University College Dublin, Belfield, Ireland; 8 Division of Pulmonary and Critical Care Medicine, University of California San Francisco, San Francisco, CA, USA; 9 J.V. Fahy and M.A. Mall contributed equally as senior authors

## Abstract

**Background:**

Mucin disulfide cross-links mediate pathologic mucus formation in muco-obstructive lung diseases. MUC-031, a novel thiol-modified carbohydrate compound, cleaves disulfides to cause mucolysis. The aim of this study was to determine the mucolytic and therapeutic effects of MUC-031 in sputum from patients with cystic fibrosis (CF) and mice with muco-obstructive lung disease (βENaC-Tg mice).

**Methods:**

We compared the mucolytic efficacy of MUC-031 and existing mucolytics (N-acetylcysteine (NAC) and recombinant human deoxyribonuclease I (rhDNase)) using rheology to measure the elastic modulus (G′) of CF sputum, and we tested effects of MUC-031 on airway mucus plugging, inflammation and survival in βENaC-Tg mice to determine its mucolytic efficacy *in vivo*.

**Results:**

In CF sputum, compared to the effects of rhDNase and NAC, MUC-031 caused a larger decrease in sputum G′, was faster in decreasing sputum G′ by 50% and caused mucolysis of a larger proportion of sputum samples within 15 min of drug addition. Compared to vehicle control, three treatments with MUC-031 in 1 day in adult βENaC-Tg mice decreased airway mucus content (16.8±3.2 *versus* 7.5±1.2 nL·mm^−2^, p<0.01) and bronchoalveolar lavage cells (73 833±6930 *versus* 47 679±7736 cells·mL^−1^, p<0.05). Twice-daily treatment with MUC-031 for 2 weeks also caused decreases in these outcomes in adult and neonatal βENaC-Tg mice and reduced mortality from 37% in vehicle-treated βENaC-Tg neonates to 21% in those treated with MUC-031 (p<0.05).

**Conclusion:**

MUC-031 is a potent and fast-acting mucolytic that decreases airway mucus plugging, lessens airway inflammation and improves survival in βENaC-Tg mice. These data provide rationale for human trials of MUC-031 in muco-obstructive lung diseases.

## Abstract 2

**MUC-031, a novel thiol-saccharide mucolytic drug, is potent and fast acting in rheology-based sputum assays and improves mucus obstruction, airway inflammation and survival in a mouse model of muco-obstructive lung disease** http://bit.ly/3Z2UIVQ

## Introduction

Pathologic mucus occludes airways to decrease airflow and cause airway infection and inflammation in multiple chronic muco-obstructive lung diseases including cystic fibrosis (CF) and COPD [1, 2]. Important mechanisms of pathologic mucus formation include increases in the concentration of mucins [3, 4] and non-mucin polymers (*e.g.* DNA), and increases in mucin disulfide crosslinks caused by oxidant acids generated by the activity of peroxidases secreted by neutrophils in chronic airway inflammation [5].

Thiol drugs have mucolytic effects because they cleave disulfide bridges between mucins through a chemical process of thiol exchange [6]. They can also scavenge reactive oxygen species to have antioxidant effects [7]. Relatively few thiol drugs have been formulated for inhaled delivery, the preferred route of administration to achieve mucolysis. The most widely used inhaled thiol drug is N-acetylcysteine (NAC), a drug whose clinical efficacy is limited by a side-effect of bronchoconstriction. Mechanisms of NAC-induced bronchoconstriction include its hyperosmolar formulation and potential for sulfite generation when cysteine is catabolised by aminotransferases [8]. Previously, we reported that carbohydrates can be functionalised with a thiol group to generate thiol-saccharide compounds with potency advantages over NAC [5]. Additionally, because aminotransferases do not act on carbohydrates, thiol-saccharides will not generate sulfite catabolites.

Airway mucus plugging is increasingly recognised as an important cause of airflow obstruction in a spectrum of chronic lung diseases beyond CF. For example, identification and scoring of mucus plugs in computed tomography lung scans from patients with COPD and asthma revealed large subgroups of patients with high mucus plug scores that associated strongly with measures of airflow obstruction [9, 10]. There are few mucolytic drug options for these patients. Although recombinant human deoxyribonuclease I (rhDNase) and hypertonic saline are effective in patients with CF [11–13], they have not shown efficacy in COPD or bronchiectasis [14–17], and there is a high unmet need for a well-tolerated and effective mucolytic for COPD and other muco-obstructive lung diseases.

MUC-031 is a novel thiol-saccharide mucolytic developed as part of an National Heart, Lung, and Blood Institute-funded translational programme project grant [18]. The efficacy of mucolytic drugs like MUC-031 can be tested *in vitro* using a cone-and-plate rheometer to quantify the elastic and viscous properties of sputum before and after drug treatment, and *in vivo* in animal models of muco-obstructive lung disease. Mice overexpressing the β-subunit of the epithelial sodium channel in their airways (βENaC-Tg) develop key features of muco-obstructive lung disease including airway mucus plugging, airway inflammation and early mortality [19–21]. Here we set out to use rheology to determine whether MUC-031 is a more potent mucolytic than rhDNase and NAC. In addition, we tested whether MUC-031 decreases airway mucus plugging and inflammation and improves survival in βENaC-Tg mice.

## Methods

Key details about experimental methods are provided herein and additional details are presented in the supplementary material.

### Synthesis of MUC-031

We have proposed thiol-modified carbohydrates (“thiol-saccharides”) as novel thiol-based mucolytics because of their efficacy and favourable physicochemical properties for inhaled delivery [5]. Carbohydrate scaffolds are natural and nontoxic and their polar nature and high aqueous solubility leads to ease of penetration into glycosylated mucin gels. In addition, the abundance of hydroxyl groups and chiral centres on carbohydrate scaffolds allows many possibilities for the introduction of a thiol group and subsequent structure–activity relationship studies. For example, we have shown that introduction of a thiol group in the 6-position of galactose generates a thiol saccharide (methyl 6-thio-6-deoxy-α-d-galactopyranoside) with better mucolytic activity than NAC [5]. Methyl 6-thio-6-deoxy-α-d-galactopyranoside and MUC-031 are part of a library of ∼30 thiol-sacccharides synthesised by the Centre for Synthesis and Chemical Biology at University College Dublin (Dublin, Ireland) using synthetic chemistry approaches similar to that previously described for methyl 6-thio-6-deoxy-α-d-galactopyranoside [5]. Additional representative structures have been disclosed in the patent literature [22, 23]. MUC-031 was chosen as the thiol-saccharide for further clinical development by the translational programme project grant investigators for reasons related to its mucolytic potency and solution stability. Once selected as the lead, large-scale synthesis of MUC-031 was done at Cascade Chemistry (Eugene, OR, USA).

### Study participants

Induced sputum was collected from healthy controls using a 12-min induction protocol described previously [24]. Spontaneously expectorated sputum samples were collected from adult patients with CF according to protocols and informed consent procedures approved by the committee on human research at the University of California, San Francisco (San Fransisco, CA, USA). Demographics and clinical characteristics of study participants are shown in table 1.

**TABLE 1:** Demographic and clinical characteristics of healthy controls and patients with cystic fibrosis (CF) who donated sputum for rheology studies

	**Healthy subjects**	**CF patients**
**Donors**	7	25
**Age, years**	44±11.1	30.8±8.7
**Female**	3 (43)	7 (28)
**FEV_1_, L**	3.44±0.73	2.2±1.13
**FEV_1_ % predicted**	95.8±6.0	54.9±21.8
**CFTR genotype**		
F508del/F508del		13 (52)
F508del/other		8 (32)
Other/other		4(16)
***Pseudomonas* infection**		
Negative		2 (8)
Intermittent		6 (24)
Chronic		17 (68)
**Pancreatic insufficiency**		23 (92)

Data are presented as n, mean±sd or n (%). FEV_1_: forced expiratory volume in 1 s; CFTR: cystic fibrosis transmembrane conductance regulator.

### Sputum rheology

**FIGURE 1:**
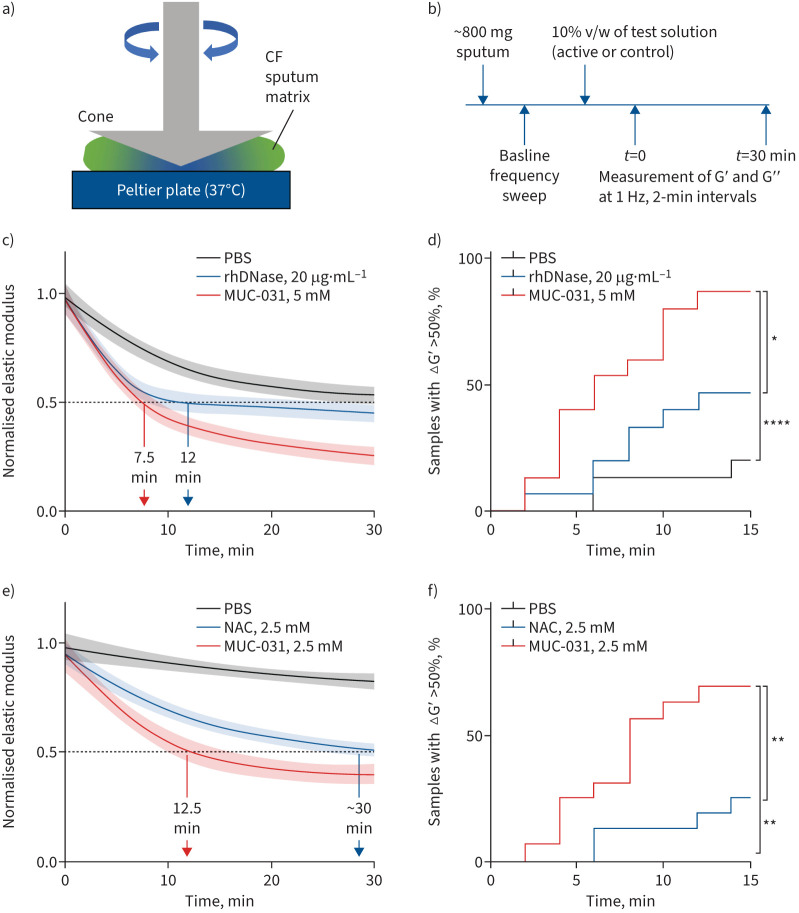
Effect of recombinant human deoxyribonuclease (rhDNase), N-acetylcysteine (NAC) and MUC-031 on the elasticity of sputum from patients with cystic fibrosis (CF). a) Schematic of a cone-and-plate rheometer. Elastic (G′) and viscous (G′′) moduli are calculated from the measured response of the samples to the oscillating angular displacement. b) Schematic of the protocol for testing the mucolytic efficacy of MUC-031, rhDNase and NAC. The G′ and G′′ moduli of CF sputum samples are measured at baseline in a frequency sweep from 0.1 to 50 Hz at 5% strain, followed by addition and manual mixing of the test agents at 10% v/w (PBS control, rhDNase, NAC and MUC-031). c) Comparison of mucolytic effect of PBS control (n=15), rhDNase (n=15, 20 µg·mL^−1^) and MUC-031 (n=15, 5 mM), in sputum not treated with protease inhibitors, as measured by change in G′ over 30 min and analysed using a generalised additive modelling (GAM) framework. d) Percentage of samples in each group (PBS, rhDNase and MUC-031) as function of time for which GAM-normalised G′ is decreased by 50%. e) Comparison of mucolytic effect of PBS control (n=8), NAC (n=16, 2.5 mM) and MUC-031 (n=16, 2.5 mM), in sputum treated with protease inhibitors Halt and EDTA, as measured by change in G′ over 30 min and analysed by GAM. f) Percentage of samples in each group (PBS, NAC and MUC-031) as function of time for which GAM-normalised G′ is decreased by 50%. In c) and e) the solid lines represent the model estimates for βj (referred to as normalised G′); the surrounding lighter shades indicate 95% pointwise confidence intervals over the time course. Nonoverlapping lines and surrounding colours are indicative of statistically significant differences between conditions. *: p<0.05, **: p<0.01, ****: p<0.0001 for comparison of cumulative curves for MUC-031 *versus* rhDNase, NAC or PBS.

The elastic (G′) and viscous (G′′) moduli of sputum were measured using a cone-and-plate rheometer (AR-2000 and DHR-2 devices; TA Instruments, New Castle, DE, USA) [5]. Detailed methods are provided in the supplementary material. Briefly, aliquots of sputum were interrogated in a strain-controlled mode by oscillating the cone geometry at 5% strain and measuring the torque. After completion of baseline measurements, the geometry was raised and test compound solution was added and mixed with the sputum. Timed oscillation measurements were taken repeatedly at 2-min intervals for ≥30 min (figure 1a and b). To accommodate baseline variability in sputum G′ and G′′, a varying-coefficient model was estimated *via* a generalised additive modelling framework (GAM). Each sample tested is treated independently by GAM, including separate aliquots from the same donor. The model generates a smooth term, β_j_, as function of time, that is referred to as normalised G′ or normalised G′′. In initial experiments we noticed that the addition of PBS to CF sputum decreased the G′ and G′′ values. Sputum samples in which the mucolytic effects of MUC-031 and NAC were compared were treated with protease inhibitor cocktail (Halt; ThermoFisher Scientific, Waltham, MA, USA) and EDTA, but sputum samples in which the mucolytic effects of MUC-031 and rhDNase were compared were not treated with protease inhibitors, because these inhibitors could decrease the activity of magnesium-/calcium-dependent rhDNase.

### Animal studies

All animal studies were approved by the animal welfare authority responsible for the Charité – Universitätsmedizin Berlin (Landesamt für Gesundheit und Soziales Berlin, Berlin, Germany). Treatment studies were performed in βENaC-Tg mice and wild-type littermates on the C57BL/6N strain background [19, 25]. MUC-031 (131 mg·mL^−1^) or vehicle alone was applied by intratracheal instillation (adult mice) or intranasal instillation (neonatal mice) in a volume of 1 µL·g^−1^ bodyweight. To determine acute and chronic treatment effects of MUC-031 in established muco-obstructive lung disease, adult βENaC-Tg and wild-type mice were treated either three times in 1 day or twice a day for 2 weeks. To determine effects of preventive treatment, neonatal βENaC-Tg and wild-type mice were treated from the first day of life twice a day for 2 weeks.

### Bronchoalveolar lavage cell counts and cytokine measurements

Bronchoalveolar lavage (BAL) was obtained and cell counts were determined, as previously described [19]. Keratinocyte chemoattractant (KC), tumour necrosis factor (TNF)-α and interleukin (IL)-13 were measured using commercially available cytometric bead array kits (BD Biosciences, San Jose, CA, USA) according to the manufacturer's instructions.

### Histology and airway morphometry

Left lungs were sectioned transversally at the level of the proximal intrapulmonary main axial airway. Our prior studies in βENaC-Tg mice showed that this airway region consistently exhibits mucus obstruction and that response to therapy with mucolytic agents in this airway region correlates with responses in distal airways [20, 26, 27]. Airway mucus content was assessed by determining the volume of Alcian blue–periodic acid–Schiff-positive material per surface area (nL·mm^−2^) of the airway, as described previously [20, 28].

### Mucin agarose gel electrophoresis

Mucin Western blotting was performed, as described previously [29, 30]. For Western blots of human sputum, we used a mouse monoclonal antibody against human MUC5B (sc-393952; Santa Cruz, Dallas, TX, USA) and a mouse monoclonal antibody against human MUC5AC (MA5-12178; Invitrogen, Waltham, MA, USA) and for Western blots of BAL supernatants from mice, we used mouse monoclonal antibody against murine MUC5B (sc21768; Santa Cruz). Details on the immunoblotting procedures are provided in the supplementary material.

### Statistical analysis

#### Analysis of rheology data

A varying-coefficient model was estimated *via* a GAM framework, as described earlier and in the supplementary material. Time points for which treatment conditions led to halving of the baseline elastic or viscous moduli of CF sputum samples were used to generate cumulative event curves and analysed for significant difference using GraphPad Prism 9.2 (GraphPad Software, San Diego, CA, USA).

#### Analysis of data from mouse studies

Data were analysed using GraphPad Prism 8.2.0 and are reported as mean±sem. Statistical analyses were performed using one-way ANOVA, two-way ANOVA, Kruskal–Wallis test and Kaplan–Meier survival analysis, as appropriate, and p<0.05 was accepted to indicate statistical significance.

## Results

### Cross-linking of mucin polymers in CF sputum is revealed by marked increases in elastic modulus

Consistent with prior reports from the present authors and others [5, 31], we found that the G′ of sputum in health is higher than its G′′ across a broad range of frequencies (supplementary figure S2a). This G′ dominance and plateau, as well as the identical dependence of G′ and G′′ on frequency (G′ and G′′ are parallel lines), are hallmarks of a cross-linked gel. In CF sputum, the predominant abnormality is a large increase in elastic response indicative of a densely cross-linked gel (supplementary figure S2a), and the G′ and G″ at a frequency of 1.0 Hz is markedly higher in CF than in health (supplementary figure S2b). Based on these data, we focused our analysis of the mucolytic effects of MUC-031 on its effects on sputum G′.

### Pre-treatment with a protease inhibitor cocktail inhibits PBS effects on the G′ of CF sputum

We found that addition of PBS decreases the G′ of CF sputum (supplementary figure S1), and based on our prior work [32], we hypothesised that this PBS effect is due to sputum protease activity. Although pre-treatment of sputum with EDTA (an inhibitor of metalloproteinases) does not inhibit this PBS effect on sputum G′, pre-treatment with Halt (which inhibits serine proteases, cysteine proteases, aspartic acid proteases and aminopeptidases), combined with EDTA significantly decreased the PBS effect (supplementary figure S1). These data suggest that proteases liberated from the mucin matrix following addition of PBS may explain its G′-lowering effects and that neutrophil serine proteases (neutrophil elastase, cathepsin G and proteinase 3) or cysteine proteases (cathepsin S and L) are the most probable protease mediators of this effect.

### MUC-031 is a more potent and faster acting mucolytic than rhDNase and NAC

To compare rhDNase and MUC-031, we tested rhDNase at 5 µg·mL^−1^ and 20 µg·mL^−1^ based on drug levels reported in sputum from treated patients with CF [33]. We found that MUC-031 (5 mM) caused a larger decrease in G′ in CF sputum than rhDNase (figure 1c) and that the mucolytic effects of 5 µg·mL^−1^ and 20 µg·mL^−1^ of rhDNase were similar (supplementary figure S4). To compare the speed of onset of mucolysis for rhDNase and MUC-031, we used two analysis approaches. First, we compared the time taken for sputum G′ to decrease by ≥50% and found that the time was shorter for MUC-031 than for rhDNase (7.5 *versus* 11.9 min; figure 1c). Second, we defined mucolysis as a 50% decline from baseline in G′ and we compared the proportion of CF sputum samples that underwent mucolysis within 15 min of addition of drug. We found that the percentage of MUC-031-treated sputum samples that underwent mucolysis by 15 min was much larger for MUC-031 than for rhDNase (87% *versus* 47%, p<0.05; figure 1d).

To compare MUC-031 and NAC, we tested both drugs at 2.5 mM because of the robust effects of MUC-031 seen at 5 mM and because we reasoned that the addition of Halt and EDTA to inhibit autolysis by proteases would increase signal-to-noise ratio. Compared to NAC, MUC-031 caused a larger decrease in G′ than NAC (figure 1e). In addition, MUC-031 had much faster speed of onset, as evidenced by a shorter time for the G′ to decrease by 50% (12.5 min for MUC-031 and ∼30 min for NAC; figure 1e) and a much larger proportion of sputum samples that underwent mucolysis by 15 min (69% for MUC-031 and 25% for NAC, p<0.01; figure 1f).

To explore dose–response effects for MUC-031 at high *versus* low levels of protease activity, we compared the effects of different drug concentrations in CF sputa pre-treated with EDTA alone or in CF sputa pre-treated with EDTA plus Halt. In CF sputa pre-treated with EDTA, we found a dose–response effect for the 2.5 mM and 5 mM MUC-031 concentrations, whereas in CF sputa pre-treated with Halt plus EDTA we found a dose–response effect for the 0.5 mM and 2.5 mM MUC-031. In these Halt plus EDTA experiments, the effect of MUC-031 at 0.5 mM was equivalent to the effect of NAC at 2.5 mM (supplementary figure S3).

In addition, we compared the effects of rhDNase, MUC-031 and NAC and PBS control on the viscous modulus (G′′) of CF sputum. The effects of the drugs on G′′ were much smaller than the effects seen for G′ (supplementary figures S5 and S6).

### MUC-031 cleaves MUC5B and MUC5AC in CF sputum

**FIGURE 2:**
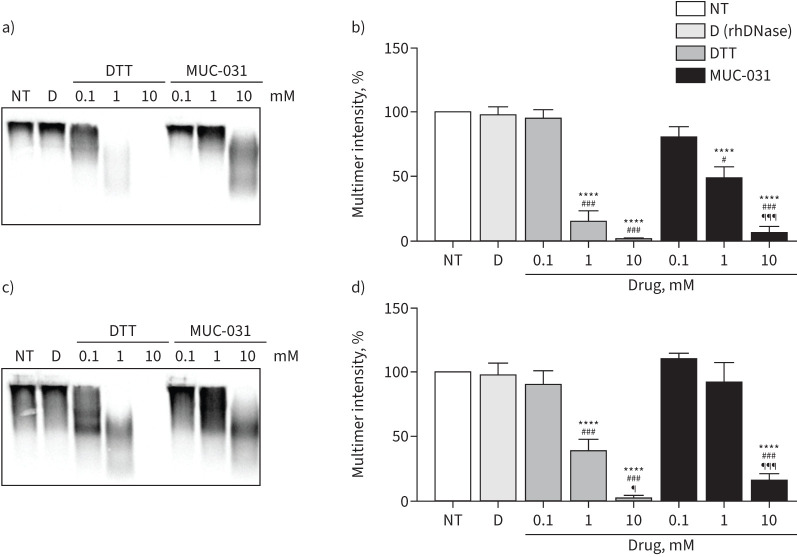
Effect of recombinant human deoxyribonuclease (rhDNase), dithiothreitol (DTT) and MUC-031 on mucin size in sputum from patients with cystic fibrosis (CF). Freshly collected sputum samples from CF patients were treated with rhDNase (20 µg·mL^−1^) or increasing concentration (0.1–10 mM) of DTT or MUC-031 at 37°C for 30 min; mucin was separated by gel electrophoresis; and Western blots were stained with antibodies against MUC5B and MUC5AC. a–d) Representative a, c) Western blots and b, d) summary of effect of rhDNase, and increasing concentrations of DTT and MUC-031 on high-molecular-weight intensity of a, b) MUC5B and c, d) MUC5AC expressed as percentage of untreated sputum aliquots (n=8–10 per group). NT: no treatment; D: rhDNase. ****: p<0.0001 compared with NT samples; ^#^: p<0.05, ^###^: p<0.001 compared with 0.1 mM concentration of same drug; ^¶^: p<0.05, ^¶¶¶^: p<0.001 compared with 1 mM concentration of same drug.

To determine if MUC-031 cleaves the major gel forming mucins in the airway (MUC5B and MUC5AC), we compared the effects of rhDNase and increasing concentrations (0.1–10 mM) of MUC-031 and dithiothreitol on the size of MUC5B and MUC5AC in Western blots of CF sputum. While rhDNase had no effect on high-molecular-weight intensity of MUC5B or MUC5AC, we found a dose-dependent reduction of multimer intensity for both mucins by MUC-031 (figure 2).

### MUC-031 decreases airway mucus plugging and airway inflammation in adult βENaC-Tg mice

**FIGURE 3:**
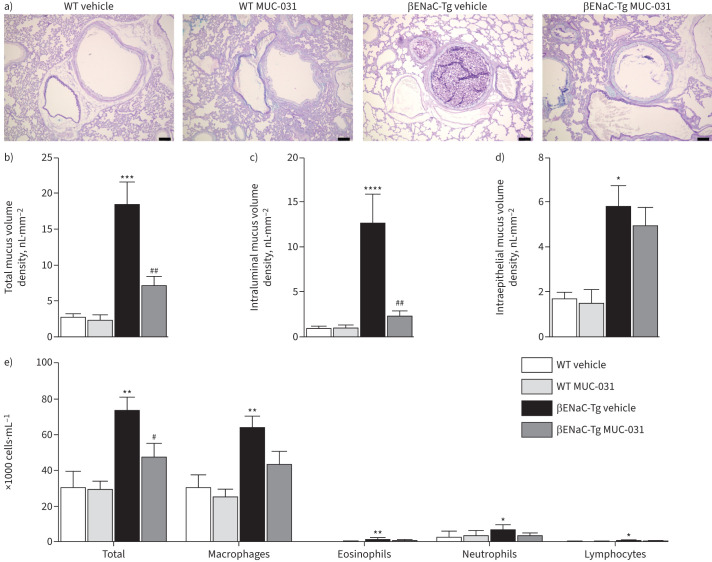
Acute treatment with MUC-031 reduces airway mucus plugging and inflammation in adult βENaC-Tg mice with chronic muco-obstructive lung disease. Adult βENaC-Tg mice and wild-type (WT) littermate controls were treated with MUC-031 or vehicle alone by intratracheal instillation three times in 1 day. a) Representative airway histology of βENaC-Tg and WT mice after acute treatment. Sections were stained with Alcian blue–periodic acid–Schiff (AB-PAS) to determine the presence of intraluminal and intraepithelial mucus. Scale bars=100 µm. b–d) Quantification of b) total, c) intraluminal and d) intraepithelial mucus content determined by measuring the volume density of AB-PAS positive material in proximal main axial airways (n=8–17 per group). e) Effects of acute treatment with MUC-031 on inflammatory cell counts in bronchoalveolar lavage (n=8–16 per group). *: p<0.05, **: p<0.01, ***: p<0.001, ****: p<0.0001 compared with vehicle-treated WT mice; ^#^: p<0.05, ^##^: p<0.01 compared with vehicle-treated βENaC-Tg mice.

To investigate the mucolytic efficacy of MUC-031 *in vivo*, we first studied the effects of MUC-031 treatment in adult βENaC-Tg mice and wild-type littermate controls in single day (acute) and then 14-day (chronic) treatment protocols. For the acute treatment protocol, MUC-031 or vehicle alone was administered by intratracheal instillation to βENaC-Tg and wild-type mice three times in 1 day, after which lung tissue and BAL were analysed for airway mucus obstruction and inflammation outcomes. Consistent with results from previous studies [20, 26], vehicle-treated adult βENaC-Tg mice showed airway mucus plugging and airway inflammation that was not evident in vehicle-treated wild-type mice (figure 3, supplementary figure S7a). Specific findings in BAL in the βENaC-Tg mice included increases in total cell number, macrophages (enlarged morphologically), neutrophils and eosinophils. Specific findings in lung tissue were increases in total and intraluminal mucus volume density. Compared to vehicle-treated βENaC-Tg mice, MUC-031-treated βENaC-Tg mice showed significant decreases in total and intraluminal mucus volume density in the airways, whereas intraepithelial mucus volume density (a measure of goblet cell metaplasia) did not change (figure 3a–d). Additionally, MUC-031-treated βENaC-Tg mice showed significant decreases in BAL total cell counts with a trend to decrease BAL macrophage and neutrophil numbers (macrophage size decreased significantly) (figure 3e, supplementary figure S7a).

**FIGURE 4:**
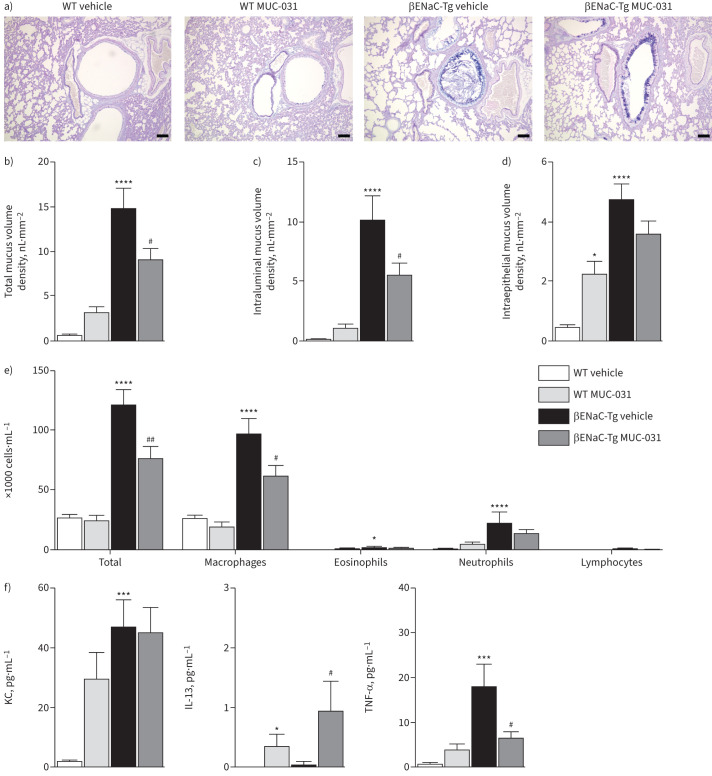
Chronic treatment with MUC-031 reduces airway mucus plugging and inflammation in adult βENaC-Tg mice with chronic muco-obstructive lung disease. Adult βENaC-Tg mice and wild-type (WT) littermates were treated with MUC-031 or vehicle alone by intratracheal instillation twice daily for 2 weeks. a) Representative airway histology of βENaC-Tg and WT mice after chronic treatment. Sections were stained with Alcian blue–periodic acid–Schiff (AB-PAS) to determine the presence of intraluminal and intraepithelial mucus. Scale bars=100 µm. b–d) Quantification of b) total, c) intraluminal and d) intraepithelial mucus content determined by measuring the volume density of AB-PAS positive material in proximal main axial airways (n=15–22 per group). e, f) Effects of e) chronic treatment with MUC-031 on inflammatory cell counts and f) concentrations of keratinocyte chemoattractant (KC), tumour necrosis factor (TNF)-α and interleukin (IL)-13 in bronchoalveolar lavage (n=10 per group). *: p<0.05, ***: p<0.001, ****: p<0.0001 compared with vehicle-treated WT mice; ^#^: p<0.05, ^##^: p<0.01 compared with vehicle-treated βENaC-Tg mice.

For the chronic treatment protocol, MUC-031 or vehicle alone was administered by intratracheal instillation twice daily for 14 days to adult βENaC-Tg mice and wild-type littermate controls. No clinical signs or symptoms suggestive of toxicity or adverse events and no deaths were observed in adult wild-type or βENaC-Tg mice during chronic treatment with MUC-031. Compared to vehicle-treated βENaC-Tg mice, MUC-031-treated mice showed significant decreases in total and intraluminal mucus volume density in the airways without any significant change in intraepithelial mucus volume density (figure 4a–d). In addition, MUC-031-treated mice showed significant decreases in BAL total cell counts, macrophages and macrophage size and a trend towards a decrease in neutrophils (figure 4e, supplementary figure S7b). Furthermore, MUC-031-treated mice showed a significant decrease in BAL levels of TNF-α and a slight increase of IL-13, without any effect on KC levels (figure 4f). Treatment of adult wild-type mice with MUC-031 twice daily for 14 days was associated with an increase in intraepithelial mucus volume density in the airways and an elevated expression of *Muc5ac* transcripts (figure 4d, supplementary figure S8a). Furthermore, chronic treatment of adult wild-type mice with MUC-031 did not significantly change BAL cells or TNF-α levels, but was associated with a slight increase of IL-13, and there was a trend for an increase in KC levels (figure 4f).

### MUC-031 improves survival in neonatal βENaC-Tg mice in context of decreased airway mucus plugging and inflammation

**FIGURE 5:**
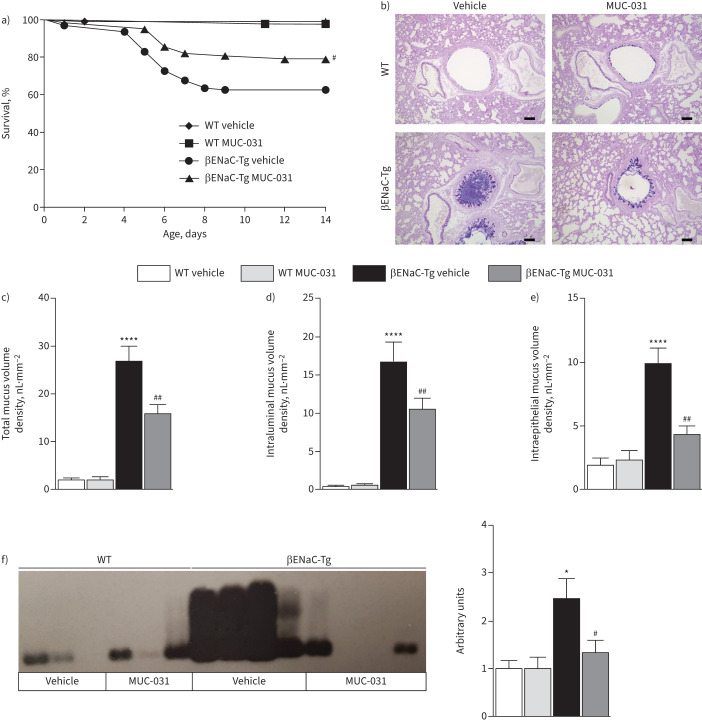
Preventive treatment with MUC-031 reduces mortality and airway mucus plugging in neonatal βENaC-Tg mice. Neonatal βENaC-Tg mice and wild-type (WT) littermates were treated with MUC-031 or vehicle alone by intranasal instillation twice daily from the first day of life for a period of 2 weeks. a) Effect of preventive treatment with MUC-031 on survival (n=49–95 per group). b) Representative airway histology of βENaC-Tg and WT mice after preventive treatment with MUC-031. Sections were stained with Alcian blue–periodic acid–Schiff (AB-PAS) to determine the presence of intraluminal and intraepithelial mucus. Scale bars=100 µm. c–e) Quantification of c) total, d) intraluminal and e) intraepithelial mucus content determined by measuring the volume density of AB-PAS-positive material in proximal main axial airways (n=14–36 per group). f) Representative agarose gel Western blots and corresponding densitometry of bronchoalveolar lavage samples stained with a murine anti-MUC5B antibody (n=23–32 per group). *: p<0.05, ****: p<0.0001 compared with vehicle-treated WT mice; ^#^: p<0.05, ^##^: p<0.01 compared with vehicle-treated βENaC-Tg mice.

βENaC-Tg mice do not have airway mucus plugging or airway inflammation at birth, but develop these pathologies shortly after birth, where they contribute to increased mortality [20, 26]. A notable feature of neonatal βENaC-Tg mice compared to adult βENaC-Tg mice is that they show features of airway type 2 inflammation [20, 26, 34–36]. We investigated whether preventive treatment of newborn βENaC-Tg mice with MUC-031 improves survival by preventing development of airway mucus plugging and airway inflammation. Neonatal βENaC-Tg and wild-type mice were treated with intranasal instillation of MUC-031 or vehicle control twice daily for 14 days from the first day of life. Similar to the chronic treatment in adult mice, no clinical signs suggestive of toxicity were observed in neonatal mice of either genotype, and no deaths were observed in wild-type mice during preventive treatment with MUC-031. Compared to vehicle-treated βENaC-Tg mice, preventive treatment with MUC-031 in βENaC-Tg mice significantly improved survival (figure 5a). This survival benefit was probably conferred because preventive MUC-031 treatment significantly decreased airway mucus plugging, as evidenced by decreases in the total and intraluminal mucus volume density in the airways of surviving βENaC-Tg mice (figure 5b–d). In addition, preventive treatment with MUC-031 in βENaC-Tg mice significantly decreased intraepithelial mucus volume density, reflecting goblet cell metaplasia (figure 5e). Furthermore, Western blotting of mucins in BAL showed partial depolymerisation of MUC5B and a decrease in MUC5B concentration (figure 5f).

**FIGURE 6:**
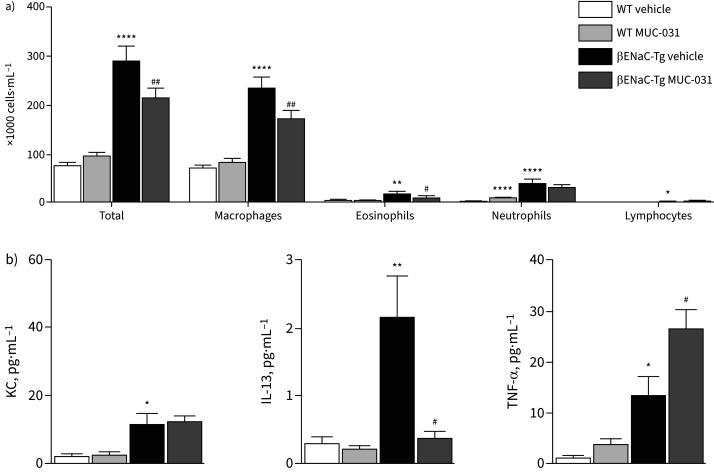
Effects of preventive treatment with MUC-031 on airway inflammation in neonatal βENaC-Tg mice. Neonatal βENaC-Tg mice and wild-type (WT) littermates were treated with MUC-031 or vehicle alone by intranasal instillation twice daily from the first day of life for a period of 2 weeks. a) Inflammatory cell counts (n=26–53 per group) and b) concentrations of keratinocyte chemoattractant (KC), tumour necrosis factor (TNF)-α and interleukin (IL)-13 (n=8–12 per group) in bronchoalveolar lavage of βENaC-Tg and WT mice after preventive treatment. *: p<0.05, **: p<0.01, ****: p<0.0001 compared with vehicle-treated WT mice; ^#^: p<0.05, ^##^: p<0.01 compared with vehicle-treated βENaC-Tg mice.

In exploring the effects of preventive treatment with MUC-031 in βENaC-Tg mice on lung inflammation, we found that MUC-031 significantly decreased BAL total cell counts, macrophages, eosinophils and macrophage size (figure 6a, b, and supplementary figure S7). MUC-031 also decreased BAL IL-13 levels, but, in contrast to findings in adult βENaC-Tg mice, increased BAL TNF-α levels (figure 6b).

## Discussion

Airway mucus plugging is an important cause of airflow obstruction and nidus for inflammation and infection in patients with a spectrum of muco-obstructive lung diseases including CF and COPD, and there are few mucolytic drug options for these patients. Here we show that a novel thiol-saccharide compound (MUC-031) is a more potent mucolytic drug than rhDNase and NAC and that its administration to βENaC-Tg mice (a model of muco-obstructive lung disease) causes beneficial effects on lung health that provide a rationale for clinical trials in humans.

Confirming prior reports [5, 32, 37], we show here that the biophysical signature of healthy airway mucus is that its elastic modulus (G′) is <1 Pa across a broad range of frequencies, and that its G′ is higher than its viscous modulus (G′′). This G′ dominance and plateau are a hallmark of a cross-linked gel. Pathologic airway mucus, occurring in a range of lung diseases from CF to COPD, is consistently characterised by large increases in G′ [5, 32, 37], indicative of a more densely cross-linked gel that is stiffer and harder for the mucociliary escalator to transport. The goal of mucolytic treatment is therefore to normalise the G′ of pathologic mucus, so as to restore the ability of the mucocilary escalator to transport it. In optimising methods to test the mucolytic effects of MUC-031 in sputum using rheology methods, we found that the addition of PBS decreased sputum G′ and that this effect was largely inhibited by protease inhibitors. Thus, hydration of pathologic mucus gels with saline may liberate proteases that have mucolytic effects, and it is important to control for these protease effects in sputum rheology studies of novel mucolytic agents.

In comparing the effects of MUC-031, rhDNase and NAC on the G′ of CF sputum, we found that MUC-031 potently and quickly decreases the G′, and that it has larger and faster-acting effects than rhDNase. This finding highlights the importance of disulfide mucin cross-linking *versus* free DNA as a mechanism of increased mucus gel elasticity in CF airway mucus. We have shown previously how DNA polymers form a shell around a densely cross-linked mucin core in CF airway mucus [5]. The potent mucolytic effect of MUC-031 shown here supports a concept in which MUC-031 cleaves disulphide-linked mucin polymers in the core of the mucus gel to transform it from its pathologic solid-like state to a more physiologic liquid-like state that is more easily cleared. The superior potency and speed of onset of MUC-031 relative to NAC may relate to the chemical advantages of thiol-saccharides that include neutral electrical charge and interactions with glycosylated mucins through hydrogen bonding. Our finding that MUC-031 lyses mucus twice as quickly as NAC is a therapeutic advantage because residence time in the airway lumen for these two inhaled drugs is likely to be <60 min [38, 39], and mucolytic drugs with faster speed of effect will be more likely to effectively lyse pathologic mucus. Irrespective of any efficacy advantages, thiol-saccharides also have advantages in terms of tolerability, because they can be formulated as iso-osmolar aerosols and, unlike NAC, they are not catabolised by aminotransferases to generate sulfites. Both of these features mean that MUC-031 is unlikely to cause bronchoconstriction as a side-effect of treatment. Additionally, thiol-saccharides are highly soluble in water and thus are well suited for inhaled delivery by a nebuliser.

We demonstrated the mucolytic efficacy of MUC-031 *in vivo* in experiments in βENaC-Tg mice that are characterised by airway mucus plugging, chronic airway inflammation and early mortality [19, 20, 36]. We used single and 14-day treatment protocols in adult mice to model treatment protocols used in management of acute exacerbations of airway disease or as maintenance treatment of chronic stable airway disease. We also included a third protocol in neonatal mice designed to determine whether MUC-031 improves survival by preventing mucus pathology and related airway inflammation in the lungs, *i.e.* this protocol models preventive treatment of pre-symptomatic stages of childhood lung diseases such as CF [40]. A single day of treatment with MUC-031 was sufficient to decrease airway mucus plugs, and 14 days of MUC-031 treatment showed sustained decreases in mucus plugs over time. Preventive treatment of newborn βENaC-Tg mice improved their survival by decreasing airway mucus pathology. Although MUC-031 consistently and effectively decreases intraluminal mucus plugs in neonatal and adult βENaC-Tg mice, we found that 2 weeks of MUC-031 treatment in adult wild-type mice caused an increase in MUC5AC gene expression and an increase in intraepithelial mucin volume. These effects did not occur after single-day treatment of adult wild-type mice with MUC-031 or 14-day treatment with MUC-031 in neonatal wild-type mice. Because we show that MUC-031 can cleave MUC5AC and MUC5B in immunoblot experiments, we speculate that cleavage of secreted or tethered mucins in the normal airway may initiate signals that upregulate MUC5AC expression in the airway epithelium. Because our murine studies only used a single dose of MUC-031, it is possible that lower doses of MUC-031 may not exert these effects on MUC5AC gene expression or intraepithelial mucin stores, but further dose response studies are needed to determine this. Taken together, these *in vivo* data demonstrate that MUC-031, delivered into the airways, effectively lyses airway mucus plugs. Our data are consistent with previous reports in βENaC-Tg mice of the mucolytic efficacy of a different experimental drug (P3001) that also cleaves mucin disulfide bridges [39]. Also relevant here are previous studies we have published showing that hypertonic saline and amiloride (an epithelial sodium channel blocker), which improve mucus hydration when delivered to the airways, also have beneficial effects in βENaC-Tg mice [26, 27, 41]. Specifically, treatment of βENaC-Tg mice with hypertonic saline decreases mucus plugs in adult mice and improves survival in neonatal mice [27], whereas treatment of βENaC-Tg mice with the epithelial sodium channel blocker amiloride decreases mucus plugging and mortality in neonatal βENaC-Tg mice, but does not decrease established airway mucus plugs in adult mice [26].

An important finding of our study is that treatment with MUC-031 has anti-inflammatory effects, including decreases total inflammatory cell numbers and cytokine levels in BAL, especially in the 14-day treatment protocols. The most likely explanation for this finding is that inhaled triggers of airway inflammation (*i.e.* bacteria, allergens and other irritants) and inflammatory cells embedded in pathologic mucus gels are cleared from the airways as the mucus is lysed and cleared, but this may not be the only mechanism. In our previous work, we found that airway mucus plugs in neonatal βENaC-Tg mice are associated with hypoxic degeneration of airway epithelial cells that causes IL-1α-mediated neutrophilic inflammation in the airway [20, 42]. And more recently, Singanayagam *et al.* [43] showed that MUC5AC has pro-inflammatory actions related to release of ATP from airway epithelial cells. Therefore, decreasing mucus plugs or airway mucins may lead to reductions in airway inflammation. Indeed, improvements in airway inflammation following improvements in mucus clearance have been demonstrated recently by Morgan *et al.* [44]. In a mouse model of asthma characterised by airway mucus plugging and eosinophilia, these authors showed that mucolytic treatment in the form of nebulised tris-2-carboxyethyl-phosphine (TCEP; 50 and 500 mM solutions) is associated with dose-dependent decreases in airway mucus plugs and a five-fold decrease in lavage eosinophil numbers with the TCEP 500 mM dose. In previous studies [20, 34], we reported that neonatal βENaC-Tg mice develop spontaneous inflammation characterised by increases in type 2 cytokines (including IL-13) and increases in eosinophils. Here we show that MUC-031 treatment prevents increases in airway IL-13 and blunts the airway eosinophil effect reported previously. In contrast to these IL-13 effects, we report an increase in TNF-α in MUC-031-treated neonatal βENaC-Tg mice. This finding did not occur in adult βENaC-Tg mice, where MUC-031 treatment reduced TNF-α levels in BAL. It is possible that MUC-031 treatment has different effects on activation of macrophage (*i.e.* a key source of TNF-α) in neonatal *versus* adult βENaC-Tg mice that could explain age-related differences in TNF-α levels in the airways, but further research is needed to determine this. Taken together, our data for the effects of MUC-031 in βENaC-Tg mice, combined with other recently published data, support an emerging concept in which pathologic mucus gels form a scaffold in which mucin rich inflammatory cell niches are sustained in airways. Disrupting these niches may have therapeutic benefits, including decreases in airway inflammation.

In summary, our data support mucolysis *via* cleavage of disulfide bonds in mucin polymers by MUC-031 as an effective strategy to reduce airway mucus plugging and inflammation in muco-obstructive lung diseases. Collectively our data support clinical development of MUC-031 as a mucolytic drug for patients with muco-obstructive lung diseases, including CF and COPD.

## Supplementary material

10.1183/13993003.02022-2022.Supp1 **Please note:** supplementary material is not edited by the Editorial Office, and is uploaded as it has been supplied by the author.
Supplementary material ERJ-02022-2022.Supplement

## Shareable PDF

10.1183/13993003.02022-2022.Shareable1 This one-page PDF can be shared freely online.
Shareable PDF ERJ-02022-2022.Shareable
