# Supplementary material for: A novel thiol-saccharide mucolytic for the treatment of muco-obstructive lung diseases
Source: Eur Respir J. 2023 May 25;61(5):2202022. doi: 10.1183/13993003.02022-2022 (PMC10209473; doi:10.1183/13993003.02022-2022)
Supplement: Supplementary file 1 [file ERJ-02022-2022.Supplement.pdf]

A novel thiol-saccharide mucolytic for the treatment of muco-obstructive lung diseases

Annalisa Addante, Wilfred Raymond, Irina Gitlin, Annabelle Charbit, Xavier Orain, Aaron Wolfe Scheffler, Aditi Kuppe, Julia Duerr, Maria Daniltchenko, Marika Drescher, Simon Y. Graeber, Anne-Marie Healy, Stefan Oscarson, John V. Fahy and Marcus A. Mall

Online Supplementary Material

Supplementay methods

Materials and reagents

The compound MUC-031 was synthesized by Centre for Synthesis and Chemical Biology, University College Dublin and by Cascade Chemistry, Inc (Eugene, OR, USA). Commercially available pharmaceutical formulations of recombinant human deoxyribonuclease (rhDNase) (Pulmozyme™, Genentech, South San Francisco, CA, USA) and N-acetylcysteine (NAC) 20% (Fresenius Kabi, Lake Zurich, IL, USA) were used in rheology studies.

Study design and participants

Sputum samples for rheology studies were collected from healthy subjects and adult patients with cystic fibrosis (CF) according to protocols and informed consent procedures approved by the Committee on Human Research at the University of California, San Francisco (UCSF). Collection of sputum samples from CF patients for mucin Western blotting was approved by the ethics committees of the Charité - Universitätsmedizin Berlin (EA2/016/18) and written consent was provided by all participants. Demographics and clinical characteristics of study participants are shown in table 1 and supplementary table E2. Non-smoking adult subjects with no history of lung disease were recruited as healthy controls using community advertising. Adult patients with CF who met the CF Foundation criteria for a diagnosis of CF (E1) were recruited from the adult CF Center at the University of California, San Francisco (UCSF). CF subjects withheld rhDNase treatment on the day of the sputum collection. Table 1 in the main text summarizes demographic and basic clinical characteristics of healthy and CF donors. For repeat CF donors, age and forced expiratory volume in the first second (FEV1) at first visit were used for calculating mean values.

Sputum collection

Healthy subjects provided a sample of induced sputum. Sputum induction involved inhalation of a nebulized solution of 3% saline for 12 minutes, as previously described (E2). CF patients

spontaneously expectorated sputum into a sputum collection cup. Any easily visible saliva layer in the sputum samples were removed by gentle pipetting prior to placement of the sample on the rheometer. The sputum samples were stored at 4ºC and most were analyzed the same day; a subset of samples were analyzed after they had been stored at 4ºC for 24 - 48 hours. Sputa used for comparative studies of MUC-031 and NAC were treated with Halt™ protease inhibitor cocktail and EDTA (ThermoFisher Scientific, Waltham, MA, USA) at 100x dilution per instructions for use immediately after collection. Sputa used for comparative studies of MUC-031 and rhDNase was not treated with Halt+EDTA due to inhibitory effects of Halt+EDTA on rhDNase activity. Sputa pretreated with EDTA only (0.5M stock, 100x dilution) was used for a sub-study of protease effects in rheology and for a subset of dose response studies.

Sputum rheology

The elastic (G') and viscous (G") moduli of sputum samples were measured using a cone and plate rheometer (AR-2000 and DHR-2 devices, TA Instruments, New Castle, DE, USA), using a Peltier plate pre-warmed to 37ºC. The rheometer provides measures of elastic modulus (G'), largely dependent on extent of cross-linking in polymer matrix, and viscous modulus (G"), largely dependent on concentration and length of polymer chains in the gel. Aliquots of sputum (~800 mg) were then interrogated in a strain-controlled mode by oscillating the cone geometry at 5% strain and measuring the torque (figure 1a). Specifically, a six step oscillatory strain sweep from 1 to 10% at 1Hz and a 15 step oscillatory frequency sweep from 0.1 to 50 Hz at 5% strain were performed with a 40 mm 2º cone geometry employing a water solvent trap. The geometry was then raised, and 89 microliters of test compound solution at 10-fold the expected final concentration were added and mixed with the sputum. Timed oscillation measurements at 1 Hz and 5% strain were then taken at 2-minute intervals over 60 minutes. Elastic or stored moduli (G') and viscous or loss moduli (G") were computed from the measured force response of the geometry oscillation on the samples. The 1 Hz measurement from the initial frequency sweep at 5% strain was used as the baseline 0-minute measurement. Mucolytic drugs (MUC-031 or rhDNase) at 10% v/w were added in droplets distributed across the surface of sputum

sample resting on Peltier plate and mixed by gentle swirling using the pipette tip; PBS was used as a control. The measurements at 1Hz / 5% strain made at successive 2 minute intervals after addition of mucolytic drug or PBS were used as outcome measures of the mucolytic efficacy of the test agents and control. Figure 1a provides a schematic illustration of the test system and figure 1b provides a schematic summary of the testing protocol. The number of independent samples tested for each test agent condition ranged from 8 to 16.

The G' and G" values of induced sputum from healthy donors ranged from 0.17 – 0.76 Pa and 0.1 to 0.26 Pa, respectively, whereas the baseline G' and G" of spontaneously expectorated sputum from CF patients ranged from 0.82 to 34.2 Pa and 0.28-9.4 Pa, respectively. Mean G' and G" baseline frequency sweeps for healthy induced sputum samples (n = 7) and CF sputum samples (n = 8) are shown in supplementary figure S2a, with individual G' and G" values at 1Hz for those samples shown in figure S2b. Because test agent effects on G' and G" depend on baseline values which vary considerably among CF sputum samples, we used a varying-coefficient model (VCM) estimated via a generalized additive modeling framework (GAM) to generate normalized G' and G" values (see statistics).

Drug dosing rationale for comparison of mucolytic drugs by rheology

For experiments with rhDNase, we targeted sputum concentrations of 5 μg/mL (equivalent to 170 nM) and 20 μg/ml (equivalent to 680 nM). The rationale for 5 μg/mL concentration came from the drug packet insert which states that mean concentration of drug in sputum after nebulization of 2.5 mg dose is 3 μg/mL (E3). The rationale for the 20 μg/mL concentration was to account for scenarios in which the volume of pathologic mucus in the airways is low or the lung deposition fraction is high (e.g. with higher efficiency nebulizers). For the MUC-031 experiments, we targeted a sputum concentration of 5 mM because this concentration is achievable when small molecules are delivered to the lungs by nebulizer. For example, a unit dose of 150 mg of MUC-031 (e.g. 3 mL of 50 mg/mL solution, MW=374 g/mol) delivered by a nebulizer with 25% efficiency to a lung mucus volume of 10 mL results in 10 mM concentration

of MUC-031 in mucus as an upper bound for achievable concentration ($[MUC\text{-}031]_{mucus}$ (mM) = Dose (mg) * 25% / MW (g/mol) / V_{mucus} (mL).

Statistics applied to the rheological measurements

Comparison of G' and G" values for healthy and CF sputum (figure S2): Comparison was performed using Mann-Whitney test assuming non-parametric distribution of the data.

Varying-coefficient model (VCM) to analyzed G' and G" data in sputum: The VCM model was estimated via a generalized additive modeling framework (GAM) (E4). In VCM-GAM, $y_{ij}(t)$ represents the rheometer measurement (e.g. G') from subject *i*, under condition *j*, at time *t*. The rheometer trajectory $y_{ij}(t)$ defined as: $y_{ij}(t) = \beta_j(t)X(t_{0,ij}) + \varepsilon_{ij}(t)$ where $X(t_{0,ij})$ is the baseline rheology value, βj is a smooth term across *t* that measures the effect of condition *j* on the rheometer trajectory as the percent decrease in the baseline rheometer measure $X(t_{0,ij})$ over time *t*, and $\varepsilon_{ij}(t) \sim N(0,\sigma^2)$ is random measurement error. Inference focuses on the smooth term βj which can be interpreted as fraction of G' (or G") remaining at time *t* across all samples tested at condition *j,* and is referred to as *normalized G'* (or *normalized G"*) in figure 1 and supplementary figure S1 and S3-S6. In this way, VCM allows rheological trajectories to be modeled as a product of condition effects and baseline rheology, allowing for a parsimonious representation which takes into account overall condition effects while accounting for baseline rheology. Each sample tested is treated independently by GAM, including separate aliquots from the same donor. Statistical inference contrasts condition effects based on their 95% pointwise confidence intervals over the time course where a lack of overlap at a fixed time point is evidence of a statistically significant difference. The model is estimated in the statistical software R (E5) using the mgcv package (version 1.8-28, R Foundation for Statistical Computing, Vienna, Austria). Smooth terms are estimated using penalized cubic regression splines with 15 knots via restricted maximum likelihood methods.

Cumulative event curves for time to 50% of starting G': Timepoints for which treatment conditions led to halving of the baseline elastic or viscous moduli of CF sputum samples were used to generate cumulative event curves and analyzed for significant difference with Prism software (Version 9.2, GraphPad Software, LLC). Curve comparisons were made applying the log rank (Mantel-Cox) test for significant difference (figures 1d and 1f).

Animal studies

All animal studies were approved by the animal welfare authority responsible for the Charité – Universitätsmedizin Berlin (Landesamt für Gesundheit und Soziales Berlin, Berlin, Germany; approval number G0045/19). The generation and genotyping of βENaC-Tg mice has been described previously (E6). Mice were housed in a specific pathogen-free animal facility and had free access to chow and water. Treatment studies were performed in hemizygous βENaC-Tg mice and wild-type littermates on the C57BL/6N genetic background (E7). MUC-031 was dissolved in citrate buffer (sodium citrate 20mM, NaCl 38.5 mM, pH 4.5). To test effects of acute treatment, 6- to 8-week-old adult βENaC-Tg mice and wild-type littermates were treated by intratracheal instillation of MUC-031 (131 mg/mL, 1µl/g body weight) or equal volumes of vehicle alone three times in one day at intervals of two hours. Two hours after the last application, mice were sacrificed, bronchoalveolar lavage (BAL) was performed and lungs were removed for histology and morphometry. To test effects of chronic treatment, four 4 week-old βENaC-Tg and wild-type mice were treated by intratracheal instillation of MUC-031 (131 mg/mL, 1µl/g body weight) or equal volumes of vehicle alone two times per day for two weeks. Twelve hours after the last treatment, mice were sacrificed for endpoint analyses. To test effects of preventive treatment, newborn βENaC-Tg and wild-type mice were treated by intranasal instillation of MUC-031 (131 mg/mL, 1µl/g body weight) or equal volumes of vehicle alone two times per day for two weeks. The concentration of MUC-031 of 131 mg/ml (or 350 mM) in the dosing solution in the murine studies was selected to ensure sufficient drug levels of MUC-031 in the airways after intratracheal (adult mice) or intranasal (neonatal mice) delivery and to balance the following factors: i) Lung dose: we aimed to ensure that we reach effective

lung doses of MUC-031, given that intratracheal and intranasal delivery in small animal models is inefficient and inhomogeneous, as shown in our previous deposition studies with other compounds, where intratracheal instillation yielded ~4% intrapulmonary deposition (E8) as well as by work of others (E9). ii) Osmolality: we aimed to avoid the use of strong hyperosmolar solutions as not to confound results with hydration effects. iii) Drug solubility: we ensured to stay below the solubility limit. Based on these considerations, we expected that dosing of mice by intratracheal or intranasal instillation of 131 mg/mL of MUC-031 results in an effective concentration in the airways of βENaC-Tg mice that is in the range of that used for our ex vivo studies on CF patient sputum. Twelve hours after the last treatment (for the two weeks treatment) or two hours after the last application (acute treatment), BAL was performed and lungs were removed for histology and further analysis. During the two week treatment period, survival was monitored daily. All analysis were performed by investigators blinded to the genotype and the treatment of the mice.

BAL cell counts, macrophage size and cytokines measurements

Mice were deeply anesthetized by intraperitoneal injection with ketamine/xylazine (160 mg /20 mg/kg body weight), the trachea cannulated, and the right lung lobes lavaged with PBS to determine total and differential cell counts, and macrophage size as previously described (E10, 11). BAL fluid samples were centrifuged at 600 x g for 5 minutes at 4°C and aliquots of cell-free supernatants were immediately stored at -80°C for further analysis. Supernatants were used to measure concentrations of KC, TNF-α and IL-13 using commercially available cytometric bead array (CBA) kits (BD Biosciences, San Jose, California, USA) according to the manufacturer's instructions.

mRNA expression analysis

Right lungs from mice were stored at 4 °C in RNAlater (Qiagen, Hilden, Germany) over night and afterwards stored at -80 °C for further analysis. Total RNA was extracted using RNeasy Mini Plus Kit (Qiagen, Hilden, Germany). RNA purity and quantity was determined using a

NanoDrop ND100 spectrophotometer (PeqLab, Erlangen, Germany). cDNA was obtained by reverse transcription of 1 µg of total RNA with High-Capacity cDNA Reverse Transcription Kit (Applied Biosystems, Waltham, MA USA). To analyze mRNA expression from the gene of interest, quantitative real-time PCR was performed on an Applied Biosystems 7500 Real Time PCR System using TaqMan universal PCR master mix and the inventoried TaqMan gene expression assays for *Muc5ac*, *Muc5b* and *Gapdh* (Applied Biosystems, Waltham, MA USA) listed in supplementary table E1 according to manufacturer's instructions. Relative fold change of target gene expression was determined by normalization to expression of the reference gene glyceraldehyde-3-phosphate dehydrogenase (*Gapdh*) (E7).

Histology and airway morphometry

Left lungs were sectioned transversally at the level of the proximal intrapulmonary main axial airway. Sections were cut at 5 µm and stained with alcian blue periodic acid-Schiff (AB-PAS). Airway mucus content was assessed by determining the volume of AB-PAS positive material per surface area (nl/mm^2) of the airway, as previously described (E10, 12). Images of airway sections were taken with an Olympus IX-71 microscope (Olympus, Hamburg, Germany) at a magnification of 10x. All morphometric measurements were performed by an investigator blinded to genotype and treatment of the mice.

Mucin agarose gel electrophoresis

Mucin Western blotting was performed using spontaneously expectorated sputum from patients with CF as previously described (E13). Briefly, fresh sputum from CF patients was diluted at 1:5 in PBS and homogenized using a 1 ml syringe and 20G cannula. Diluted sputum was treated with rhDNase 20 µg/ml (equivalent to 680 nM) or increasing concentration (0.1 – 10 mM) of MUC-031 or DTT at 37 °C for 30 minutes and then quenched with 200 mM iodoacetamide (Merck KGaA, Darmstadt, Germany) and then loaded in the agarose gel. Agarose gel electrophoresis using 0.8% agarose was combined with transfer onto a nitrocellulose membrane via vacuum. After loading the gels, proteins were separated at 80 V

(1.5 hour) with Tris-acetate-EDTA/SDS buffer. For an efficient mucin transfer, the gel was reduced for 20 minutes in a solution containing 10 mM dithiothreitol (DTT) and proteins were then transferred by vacuum blotting (MP Biomedicals, Irvine, CA, USA) to nitrocellulose membranes. Blots were probed with a mouse monoclonal antibody against human MUC5B (sc-393952, Santa Cruz, Dallas, TX, USA) diluted at 1:1000 in 1% milk-PBS and mouse monoclonal antibody against human MUC5AC (MA5-12178, Thermo Fisher Scientific, Waltham, MA, USA) diluted at 1:1000 in 1% milk-PBS. The secondary antibody was a goat anti-mouse immunoglobulin/HRP (P0047, Dako, Glostrup, Denmark), diluted 1:5000 in 1% milk-PBS. Detection was performed using OPTIMAX X-Ray Film Processor (PROTEC Medizintechnik GmbH & Co. KG, Oberstenfeld, Germany) and analysis of specific signals were carried out as previously described (E14). Mucin Western blots of BAL supernatants from mice were performed using the same protocol with some small modifications. Specifically, total protein concentration of BAL fluid was determined using Pierce™ BCA Protein Assay Kit according to the manufacturer’s instructions (Thermo Fisher Scientific, Waltham, MA, USA) and was used to control for equivalent loading. Blots were probed with a mouse monoclonal antibody against murine Muc5b (sc21768, Santa Cruz, Dallas, TX, USA) diluted at 1:1000 in 1% milk-PBS.

Statistical analysis

Data were analyzed with GraphPad Prism 8.2.0 (GraphPad Software, San Diego, USA) and are reported as mean ± SEM unless indicated otherwise. Normal distribution of data was assessed prior to statistical analysis. Statistical analyses of groups with normally distributed data sets was performed with one-way ANOVA followed by Tukey’s post hoc test or two-way ANOVA. Not normally distributed data were analyzed by Kruskal-Wallis test with Dunn's multiple comparisons test. Survival was compared using Kaplan-Meier survival analysis the log rank test. $P < 0.05$ was accepted to indicate statistical significance.

Supplementary tables

Table E1. List of TaqMan gene expression assays used for transcript analyses

Target gene	Taqman ID
Muc5b	Mm00466391_m1
Muc5ac	Mm01276718_m1
Gapdh	Mm99999915_g1

Table E2. Demographic and clinical characteristics of patients with cystic fibrosis who donated sputum for mucin Western blotting

	**CF patients**<br>Mean ± SD or n (%)
Number of donors	13
Age, years	28.1 ± 6.3
Sex, female	5 (38%)
FEV1 % predicted	44.8 ± 30.3
CFTR genotype	
F508del/F508del	8 (62%)
F508del/other	5 (38%)
Other/other	0 (0%)
Pseudomonas Infection	
Negative	6 (46%)
Intermittent	0 (0%)
Chronic	7 (54%)
Pancreatic Insufficiency	13 (100%)

Definition of abbreviations: FEV1 = forced expiratory volume in one second; SD = standard deviation.

Supplementary figures

Supplementary figure S1

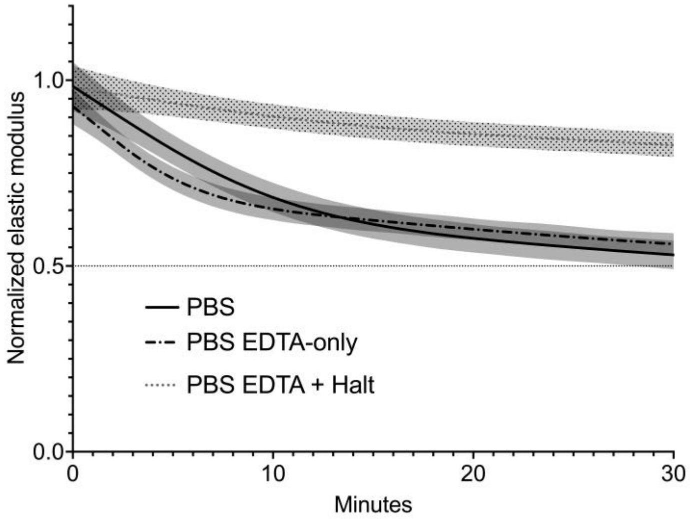

Supplementary figure S1. Effects of protease inhibitors (EDTA and Halt) on the decline in the normalized elastic modulus (G’) of cystic fibrosis (CF) sputum after addition of PBS. Untreated (with Halt or EDTA) sputum samples to which PBS was added at 10% v/w show a relatively large time-dependent decline in normalized elastic modulus (G’). Pretreatment with EDTA alone had no effect, but pretreatment with EDTA and Halt effectively inhibited the decline in G’ observed in CF sputum samples after adding PBS. n = 16 for untreated (i.e. no protease inhibitor added) sputum samples; n = 21 for EDTA-only treated samples; n = 8 for EDTA+Halt treated samples.

Supplementary figure S2

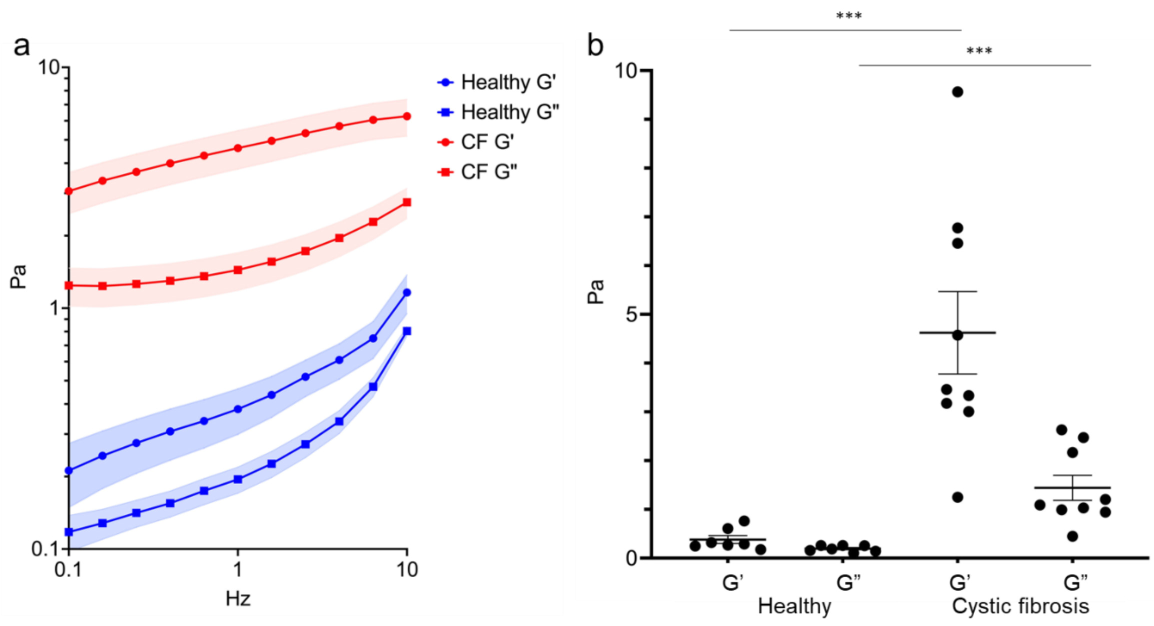

Supplementary figure S2: Comparison of the elastic and viscous behaviour of sputum in health and in cystic fibrosis (CF). a) Mean frequency sweeps of the elastic modulus (G′) and viscous modulus (G″) of induced sputum in health (open symbols, n = 7) and sputum spontaneously expectorated by patients with cystic fibrosis (CF) (closed symbols, n = 8). Error bands are SEM. In healthy sputum, the G’ predominates over the G” across a broad range of frequencies. The G’ predominance and plateau as well as the identical dependence of G’ and G” on frequency (G’ and G” are parallel lines) are hallmarks of a cross-linked gel. In CF sputum, the elastic and viscous moduli are higher than normal and the predominant abnormality is the markedly increased elastic response. b) Individual G′ and G″ (at a frequency of 1.0 Hz) in induced sputum from 7 healthy donors and spontaneously expectorated sputum from 8 CF donors. ****P* < 0.001 for G’ and G” values for healthy versus CF sputum.

Supplementary figure S3

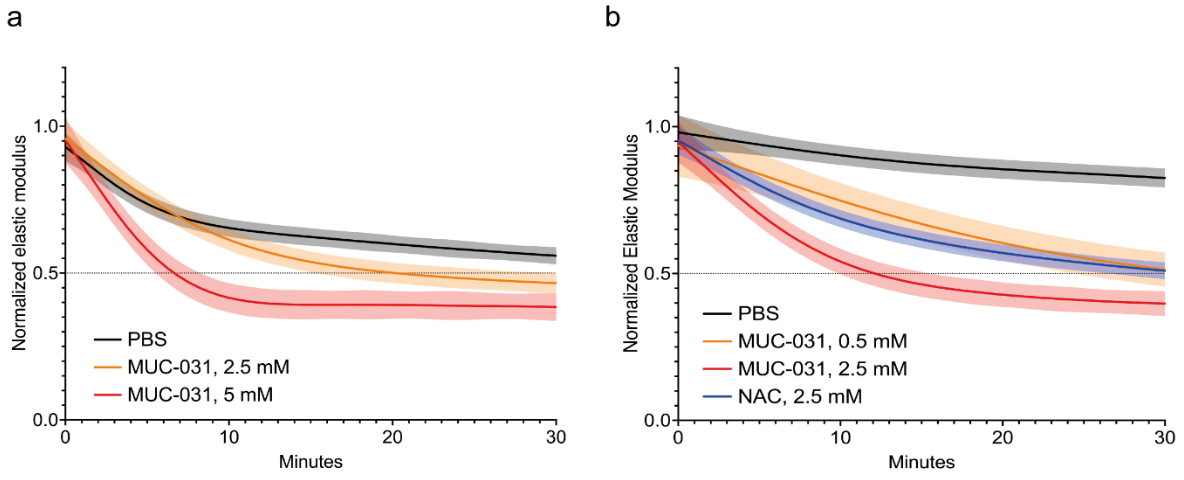

Supplementary figure S3. Effects of increasing concentrations of MUC-031 on the normalized elastic modulus (G') of sputum samples from patients with cystic fibrosis (CF). Measurements were performed after pretreatment of sputum samples with EDTA alone (a) and with a protease inhibitor cocktail of EDTA+Halt (b). Data show a dose response effect between 2.5 and 5 mM concentrations of MUC-031 in EDTA-only treated CF sputa and between 0.5 and 2.5 mM in sputa pretreated with Halt+EDTA. An overlay of NAC effects at 2.5 mM suggest that it is equivalent to the effects of 0.5 mM MUC-031. In (a), n = 21 for PBS control; n = 10 for MUC-031 at 2.5 mM; n = 13 for MUC-031 at 5 mM. In (b), n = 8 for PBS control; n = 5 for MUC-031 at 0.5 mM, n = 16 for MUC-031 and NAC at 2.5 mM condition.

Supplementary figure S4

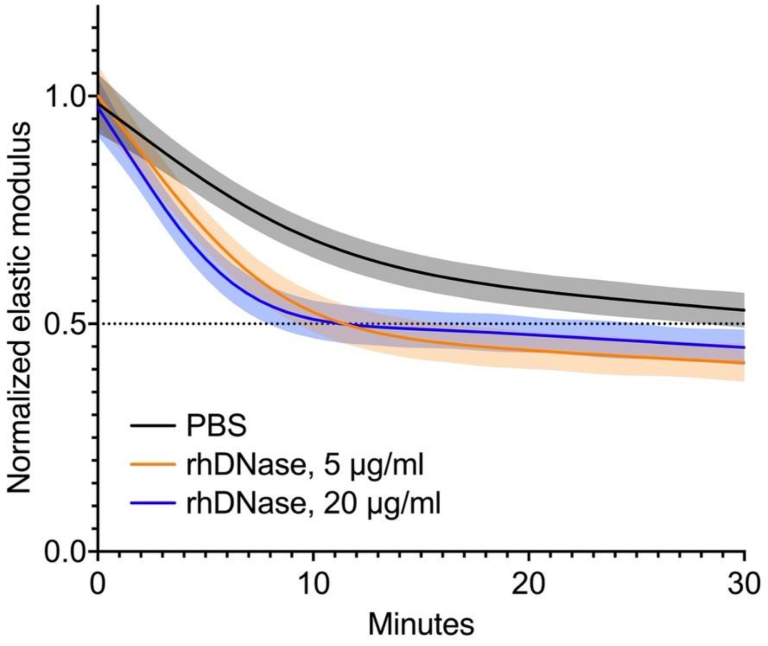

Supplementary figure S4. Effect of low dose and high dose recombinant human deoxyribonuclease I (rhDNase) on the normalized elastic modulus (G’) of cystic fibrosis sputum. The data show a lack of a dose response effect, i.e. the overlapping surround colors that show the 95% pointwise confidence intervals over the time course indicate that the differences between conditions are not statistically significant. n = 15 for all groups.

Supplementary figure S5

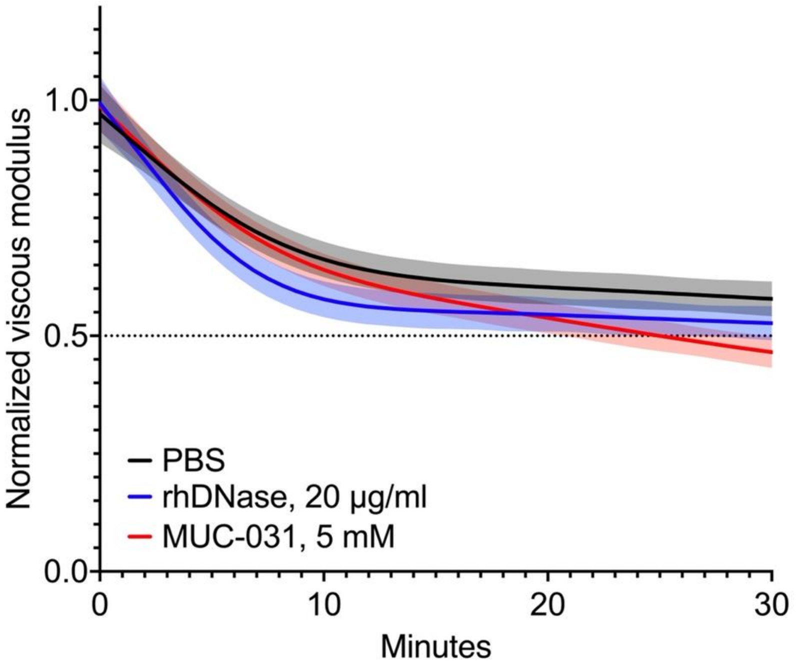

Supplementary figure S5. Effect of MUC-031 and recombinant human deoxyribonuclease I (rhDNase) and the normalized viscous modulus (G”) of cystic fibrosis sputum. The data show little difference in the effects of MUC-031 and rhDNase - i.e. the surround colors that show the 95% pointwise confidence intervals over the time course indicate only small differences between drugs. n = 15 for all groups.

Supplementary figure S6

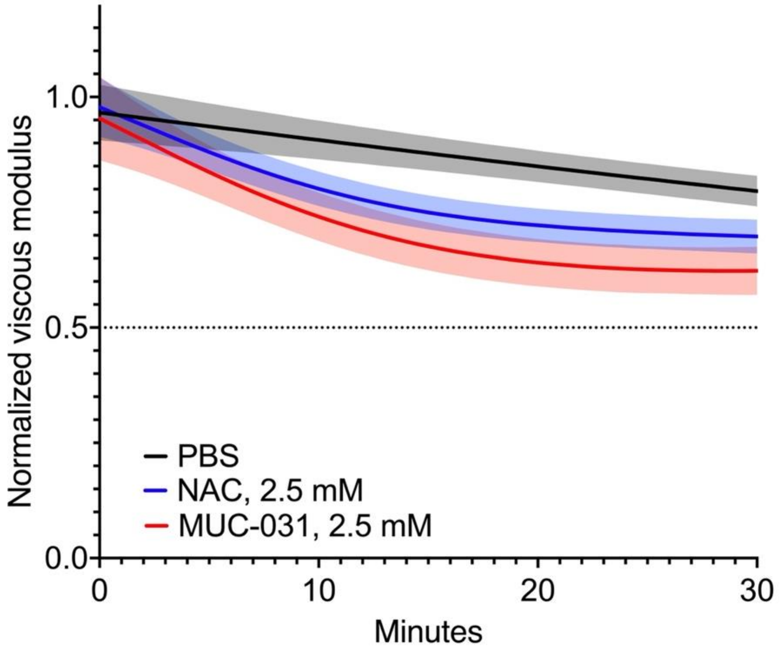

Supplementary figure S6. Effect of MUC-031 and N-acetyl cysteine (NAC) and the normalized viscous modulus (G") of CF sputum. The data show that the effect of MUC-031 on G" is larger than the effect of NAC. n = 16 for NAC and MUC-031 groups; n = 8 for PBS control.

Supplementary figure S7

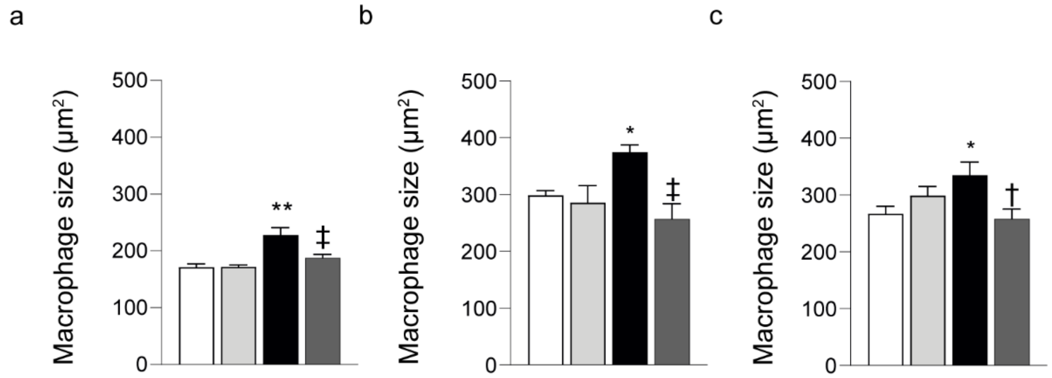

Supplementary figure S7. Effect of MUC-031 treatment on macrophage size in bronchoalveolar lavage of βENaC-Tg mice. Adult and neonatal βENaC-Tg mice and wild-type (WT) littermates were treated with MUC-031 or vehicle alone as detailed in the online supplement. a, b) Effect of acute (a) and chronic (b) treatment with MUC-031 on macrophage size in adult βENaC-Tg and WT mice (n = 6 - 9 per group). c) Effect of preventive treatment with MUC-031 on macrophage size in neonatal βENaC-Tg and WT mice (n = 13 per group). *P < 0.05 and **P < 0.001 versus vehicle-treated WT mice. $^{\dagger}P$ < 0.05 and $^{\ddagger}P$ < 0.01 versus vehicle-treated βENaC-Tg mice.

Supplementary figure S8

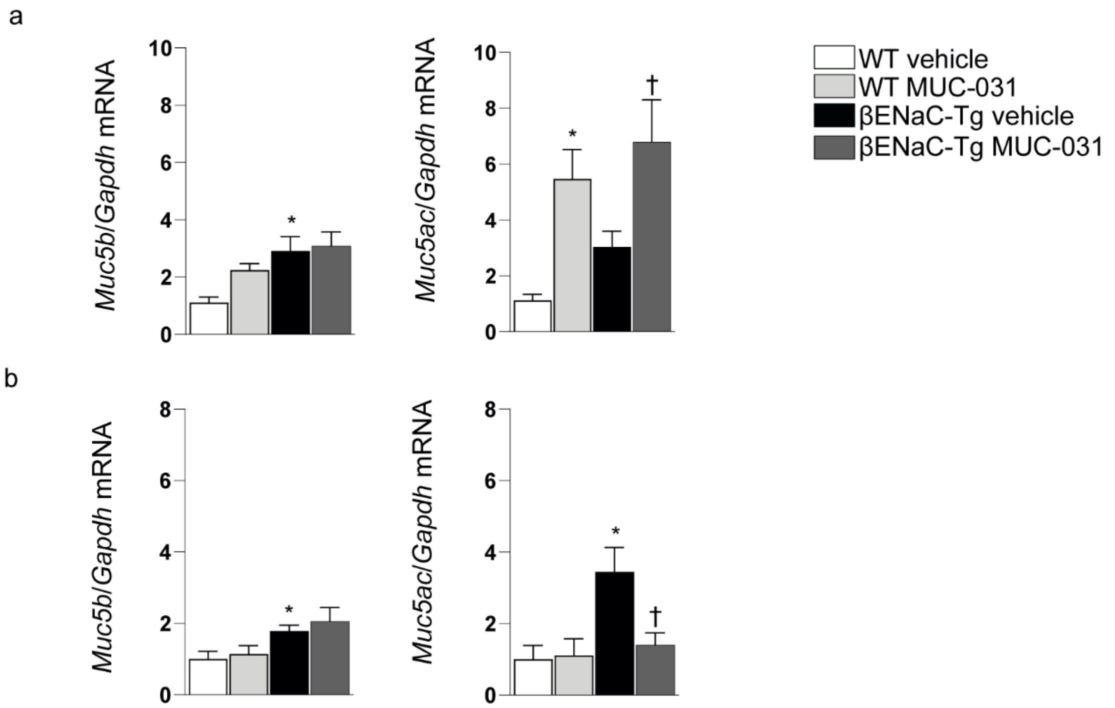

Supplementary figure S8. Expression levels of *Muc5b* and *Muc5ac* in lung tissue of wild-type (WT) and βENaC-Tg mice after treatment with MUC-031. Adult and neonatal βENaC-Tg mice and WT littermates were treated with MUC-031 or vehicle alone for two weeks. a) *Muc5b* and *Muc5ac* transcript levels in lungs of adult (a; n = 8 - 13 per group) and neonatal (b; n = 9 - 29 per group) WT and βENaC-Tg mice after treatment with MUC-031 or vehicle alone for two weeks. *$P < 0.05$ compared with vehicle-treated WT mice. $^{\dagger}P < 0.05$ compared with vehicle-treated βENaC-Tg mice.

Supplemental references

E1. Farrell PM, White TB, Ren CL, Hempstead SE, Accurso F, Derichs N, Howenstine M, McColley SA, Rock M, Rosenfeld M, Sermet-Gaudelus I, Southern KW, Marshall BC, Sosnay PR. Diagnosis of Cystic Fibrosis: Consensus Guidelines from the Cystic Fibrosis Foundation. *J Pediatr* 2017; 181S: S4-S15 e11.

E2. Yuan S, Hollinger M, Lachowicz-Scroggins ME, Kerr SC, Dunican EM, Daniel BM, Ghosh S, Erzurum SC, Willard B, Hazen SL, Huang X, Carrington SD, Oscarson S, Fahy JV. Oxidation increases mucin polymer cross-links to stiffen airway mucus gels. *Sci Transl Med* 2015; 7: 276ra227.

E3. Genentech. PULMOZYME® (dornase alfa) inhalation solution, for inhalation use. 2021; https://www.gene.com/download/pdf/pulmozyme_prescribing.pdf.

E4. Wood SN. Generalized Additive Models: An Introduction with R. 2017.

E5. Team RC. R: A Language and Environment for Statistical Computing. 2019.

E6. Mall M, Grubb BR, Harkema JR, O'Neal WK, Boucher RC. Increased airway epithelial Na+ absorption produces cystic fibrosis-like lung disease in mice. *Nat Med* 2004; 10: 487-493.

E7. Johannesson B, Hirtz S, Schatterny J, Schultz C, Mall MA. CFTR regulates early pathogenesis of chronic obstructive lung disease in betaENaC-overexpressing mice. *PLoS One* 2012; 7: e44059.

E8. Zhou Z, Treis D, Schubert SC, et al. Preventive but not late amiloride therapy reduces morbidity and mortality of lung disease in βENaC-overexpressing mice. *Am J Respir Crit Care Med* 2008: 178(12): 1245-1256.

E9. Miller MA, Stabenow JM, Parvathareddy J, et al. Visualization of murine intranasal dosing efficiency using luminescent Francisella tularensis: effect of instillation volume and form of anesthesia. *PLoS One* 2012: 7(2): e31359.

E10. Mall MA, Harkema JR, Trojanek JB, Treis D, Livraghi A, Schubert S, Zhou Z, Kreda SM, Tilley SL, Hudson EJ, O'Neal WK, Boucher RC. Development of chronic bronchitis and

emphysema in beta-epithelial Na+ channel-overexpressing mice. *Am J Respir Crit Care Med* 2008; 177: 730-742.

E11. Graeber SY, Zhou-Suckow Z, Schatterny J, Hirtz S, Boucher RC, Mall MA. Hypertonic saline is effective in the prevention and treatment of mucus obstruction, but not airway inflammation, in mice with chronic obstructive lung disease. *Am J Respir Cell Mol Biol* 2013; 49: 410-417.

E12. Harkema JR, Plopper CG, Hyde DM, St George JA. Regional differences in quantities of histochemically detectable mucosubstances in nasal, paranasal, and nasopharyngeal epithelium of the bonnet monkey. *J Histochem Cytochem* 1987; 35: 279-286.

E13. Ramsey KA, Rushton ZL, Ehre C. Mucin Agarose Gel Electrophoresis: Western Blotting for High-molecular-weight Glycoproteins. *J Vis Exp* 2016.

E14. Balazs A, Millar-Buchner P, Mulleder M, Farztdinov V, Szyrwiel L, Addante A, Kuppe A, Rubil T, Drescher M, Seidel K, Stricker S, Eils R, Lehmann I, Sawitzki B, Rohmel J, Ralser M, Mall MA. Age-Related Differences in Structure and Function of Nasal Epithelial Cultures From Healthy Children and Elderly People. *Front Immunol* 2022; 13: 822437.
