# Supplementary material for: A novel thiol-saccharide mucolytic for the treatment of muco-obstructive lung diseases
Source: Eur Respir J. 2023 May 25;61(5):2202022. doi: 10.1183/13993003.02022-2022 (PMC10209473; doi:10.1183/13993003.02022-2022)
Supplement: Supplementary file 2 [file ERJ-02022-2022.Shareable.pdf]

SHAREABLE PDF

A novel thiol-saccharide mucolytic for the treatment of muco-obstructive lung diseases

Annalisa Addante[1,2], Wilfred Raymond[3], Irina Gitlin[3], Annabelle Charbit[3], Xavier Orain[3], Aaron Wolfe Scheffler[4], Aditi Kuppe[1,2], Julia Duerr[1,2], Maria Daniltchenko[1], Marika Drescher[1], Simon Y. Graeber[1,2,5], Anne-Marie Healy[6], Stefan Oscarson[7], John V. Fahy[3,8,9] and Marcus A. Mall[1,2,5,9]

[1]Department of Pediatric Respiratory Medicine, Immunology and Critical Care Medicine, Charité – Universitätsmedizin Berlin, corporate member of Freie Universität Berlin and Humboldt-Universität zu Berlin, Berlin, Germany. [2]German Centre for Lung Research (DZL), associated partner, Berlin, Germany. [3]Cardiovascular Research Institute, University of California San Francisco, San Francisco, CA, USA. [4]Department of Epidemiology and Biostatistics, University of California San Francisco, San Francisco, CA, USA. [5]Berlin Institute of Health (BIH) at Charité – Universitätsmedizin Berlin, Berlin, Germany. [6]School of Pharmacy and Pharmaceutical Sciences, Trinity College Dublin, Dublin, Ireland. [7]Centre for Synthesis and Chemical Biology, University College Dublin, Belfield, Ireland. [8]Division of Pulmonary and Critical Care Medicine, University of California San Francisco, San Francisco, CA, USA. [9]J.V. Fahy and M.A. Mall contributed equally as senior authors.

Corresponding author: Marcus A. Mall (marcus.mall@charite.de)

Check for updates

Shareable abstract (@ERSpublications)
MUC-031, a novel thiol-saccharide mucolytic drug, is potent and fast acting in rheology-based sputum assays and improves mucus obstruction, airway inflammation and survival in a mouse model of muco-obstructive lung disease http://bit.ly/3Z2UIVQ

Cite this article as: Addante A, Raymond W, Gitlin I, *et al*. A novel thiol-saccharide mucolytic for the treatment of muco-obstructive lung diseases. *Eur Respir J* 2023; 61: 2202022 [DOI: 10.1183/13993003.02022-2022].

This single-page version can be shared freely online.

Copyright ©The authors 2023.

This version is distributed under the terms of the Creative Commons Attribution Licence 4.0.

This article has an editorial commentary: https://doi.org/10.1183/13993003.00619-2023

Received: 19 Oct 2022
Accepted: 13 March 2023

Abstract

Background Mucin disulfide cross-links mediate pathologic mucus formation in muco-obstructive lung diseases. MUC-031, a novel thiol-modified carbohydrate compound, cleaves disulfides to cause mucolysis. The aim of this study was to determine the mucolytic and therapeutic effects of MUC-031 in sputum from patients with cystic fibrosis (CF) and mice with muco-obstructive lung disease (βENaC-Tg mice).

Methods We compared the mucolytic efficacy of MUC-031 and existing mucolytics (N-acetylcysteine (NAC) and recombinant human deoxyribonuclease I (rhDNase)) using rheology to measure the elastic modulus (G′) of CF sputum, and we tested effects of MUC-031 on airway mucus plugging, inflammation and survival in βENaC-Tg mice to determine its mucolytic efficacy *in vivo*.

Results In CF sputum, compared to the effects of rhDNase and NAC, MUC-031 caused a larger decrease in sputum G′, was faster in decreasing sputum G′ by 50% and caused mucolysis of a larger proportion of sputum samples within 15 min of drug addition. Compared to vehicle control, three treatments with MUC-031 in 1 day in adult βENaC-Tg mice decreased airway mucus content (16.8±3.2 *versus* 7.5±1.2 $nL \cdot mm^{-2}$, $p<0.01$) and bronchoalveolar lavage cells (73 833±6930 *versus* 47 679±7736 $cells \cdot mL^{-1}$, $p<0.05$). Twice-daily treatment with MUC-031 for 2 weeks also caused decreases in these outcomes in adult and neonatal βENaC-Tg mice and reduced mortality from 37% in vehicle-treated βENaC-Tg neonates to 21% in those treated with MUC-031 ($p<0.05$).

Conclusion MUC-031 is a potent and fast-acting mucolytic that decreases airway mucus plugging, lessens airway inflammation and improves survival in βENaC-Tg mice. These data provide rationale for human trials of MUC-031 in muco-obstructive lung diseases.

BY
